# Caregiver Weight Misperception and Feeding Practices in U.S. Preschool-Aged Children: A Theory-Based Cross-Sectional Study

**DOI:** 10.3390/nu18121880

**Published:** 2026-06-11

**Authors:** Qutaibah Oudat, Sarah Messiah, Tamilyn Bakas, Alia Ghoneum

**Affiliations:** 1 Department of Biobehavioral Health and Nursing Science, College of Nursing, University of South Carolina, Columbia, SC 29208, USA; 2 Department of Epidemiology, Peter O’Donnell Jr. School of Public Health, University of Texas Southwestern Medical Center, Dallas, TX 75390, USA; sarah.messiah@utsouthwestern.edu; 3 Department of Population Health, College of Nursing, University of Cincinnati, Cincinnati, OH 45221, USA; bakastn@ucmail.uc.edu; 4 Department of Family Medicine, East Carolina University, Greenville, NC 27834, USA; ghoneuma23@ecu.edu

**Keywords:** weight misperception, feeding practices, preschool children, caregivers, childhood obesity, CFPQ

## Abstract

Background/Objectives: Caregiver perception of child weight may be a cognitive antecedent of feeding practices, yet evidence linking misperception to feeding practices remains inconsistent. Guided by Social Cognitive Theory, this study examined whether caregiver weight perception (underestimation, overestimation, or accurate) is independently associated with feeding practices among caregivers of U.S. preschool-aged children. Methods: Primary caregivers of children aged 3–5 years (analytic n = 139) were recruited across the Midwestern United States from April 2022 to March 2023. Weight perception was classified as accurate, underestimation, or overestimation by comparing perceived with CDC BMI-for-age category. Four Comprehensive Feeding Practices Questionnaire (CFPQ) subscales were assessed: pressure to eat, restriction for health, restriction for weight control, and monitoring. Multivariable linear regression estimated associations, adjusting for child and caregiver characteristics and child BMI-for-age z-score. Results: Overall, 45.3% of caregivers accurately perceived their child’s weight, 44.6% underestimated it, and 10.1% overestimated it. In adjusted models, underestimation was independently associated with lower restriction for health (B = −0.62, 95% CI: −1.10, −0.13, *p* = 0.013) and lower restriction for weight control (B = −0.30, 95% CI: −0.58, −0.02, *p* = 0.033) relative to accurate perception. Overestimation was marginally associated with higher restriction for weight control (B = 0.53, 95% CI: 0.00, 1.07, *p* = 0.050). No associations were observed with pressure to eat or monitoring. Conclusions: Weight misperception was selectively associated with restrictive feeding. This identifies it as a candidate cognitive target in early obesity prevention research. Longitudinal research is needed to establish causality.

## 1. Introduction

Childhood obesity remains a persistent public health concern in the United States, with prevalence among youth aged 2–19 years rising numerically from 21.2% before the COVID-19 pandemic to 22.6% during the pandemic [1]. The preschool years are a key period when eating habits form and may track into later childhood, increasing long-term obesity risk and related cardiometabolic conditions [2]. Young children rely heavily on caregivers for food provision, portioning, and mealtime structure. As a result, caregiver feeding practices are key modifiable influences on early dietary behaviors. These practices include pressure to eat, restriction, and monitoring [3,4].

Caregiver perception of child weight status may be an important cognitive antecedent of feeding practices. Prior studies suggest that caregivers who perceive their child as underweight tend to report greater pressure to eat and lower restriction, whereas those who perceive their child as overweight tend to report greater restriction or covert control [5,6]. Evidence directly examining misperception also suggests that inaccurate parental classification of child weight may be associated with feeding practices, including pressure to eat and restriction [7]. Studies indicate that approximately 30–40% of caregivers misperceive their child’s weight status, with underestimation being the predominant form [8,9]. This pattern has been linked to social desirability bias and parental reluctance to acknowledge excess child weight [8]. Empirical evidence linking weight misperception to feeding practices remains inconsistent. Several studies focus on perceived weight category, weight-related concern, or dissatisfaction rather than perception accuracy itself. For example, concern about child weight is consistently associated with greater restrictive feeding, yet its relationship with pressure to eat is less clear [6,10]. A narrative review of recent evidence concluded that parental misperceptions may influence child eating behavior and weight-control strategies, with overestimation generally linked to restriction and underestimation linked to pressure to eat, although stronger longitudinal evidence is still needed [11].

The relationship between weight misperception and feeding practices may also be confounded by caregiver and child characteristics. Some studies reported that racial/ethnic background, socioeconomic position, and parental education are associated with caregiver misperception of child weight status [8,12,13]. These associations may reflect cultural norms around body size and differential access to weight-related health information. Additionally, meta-analytic and cross-sectional evidence consistently show that parents of children with overweight or obesity frequently underestimate their child’s weight status [12,14,15]. These same caregiver and child characteristics are also associated with feeding practices, including pressure to eat and restriction [16,17]. Therefore, sociodemographic characteristics and child BMI may act as shared upstream determinants of both perception accuracy and feeding behavior and should be accounted for in analytic models. However, prior studies vary in whether they examine perceived weight status, weight-related concern, dissatisfaction, or perception accuracy, and these related constructs are sometimes discussed in ways that blur their conceptual distinction [10,11]. This makes it difficult to determine whether feeding practices are associated with misclassification of child weight status, concern about weight, or both. This distinction underscores the need to examine perception accuracy directly when evaluating associations between caregiver cognition and feeding practices.

Guided by a conceptual model derived from the Social Cognitive Theory (SCT) [18], this study positioned caregiver weight perception as a cognitive factor, feeding practices as a behavioral factor, and characteristics of caregivers and children as environmental and personal factors. The aim was to examine whether caregiver weight perception (underestimation, overestimation, or accurate perception) is independently associated with four feeding practice subscales (pressure to eat, restriction for health, restriction for weight control, and monitoring) among primary caregivers of U.S. preschoolers, after adjustment for characteristics of caregivers and children. We hypothesized that primary caregivers who underestimated their child’s weight would report lower use of weight-related restrictive practices than those with accurate perceptions, whereas overestimation would be associated with greater use of restrictive and monitoring practices. Findings were intended to clarify whether correcting weight perception is a sufficient lever for changing non-responsive feeding behavior in early childhood obesity prevention.

## 2. Materials and Methods

### 2.1. Study Design, Conceptual Model, and Participants

**Figure 1:**
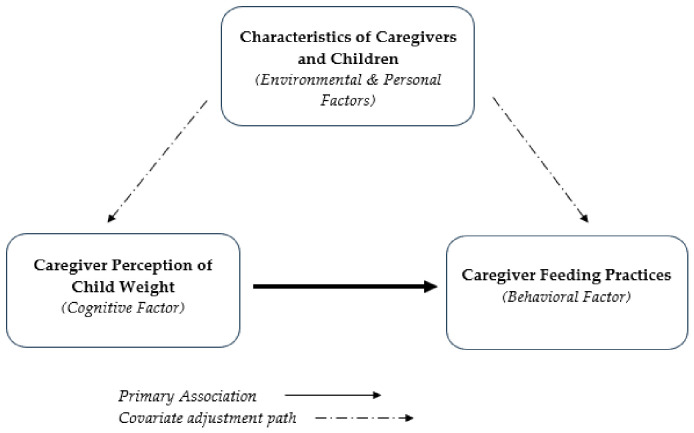
Conceptual model guided by SCT: characteristics of caregivers and children (environmental and personal factors), caregiver weight perception (cognitive factor), and feeding practices (behavioral factor).

This cross-sectional study presents additional analyses of data collected as part of a study that originally aimed at examining predictors of diet quality in preschool-aged children [19]. Guided by a framework derived from the SCT (Figure 1), we conceptualized caregiver weight perception as a cognitive factor, feeding practices as behavioral factors, and caregiver and child sociodemographic and anthropometric characteristics as personal/environmental factors. Within this framework, we treated misperception of weight status as a specific cognitive appraisal that may be associated with less responsive feeding practices after accounting for child BMI and sociodemographic context. The SCT-guided model informed our a priori selection of covariates and our primary hypotheses that underestimation would be associated with lower use of restrictive practices, whereas overestimation would be associated with greater restriction and monitoring.

The objective of the present study was to examine the association between caregiver weight misperception and feeding practices of preschool-aged children (3–5 years). Data were collected between April 2022 and March 2023 from primary caregivers of preschool-aged children (3–5 years). Participants were recruited using a combination of convenience and snowball sampling methods. Recruitment occurred through a Facebook advertising campaign and through community settings across the Midwestern United States. These settings included daycare and early childhood education centers and community religious institutions (mosques, churches, and synagogues). Eligible caregivers were (1) the primary caregiver of a child aged 3–5 years, (2) residing with the child at least 50% of the time, (3) English-literate, and (4) U.S. residents. Surveys were completed via a secure REDCap link or mailed study package. The analytic sample (n = 146) was powered for the parent study using Green’s regression formula [19]; no separate power calculation was conducted for the present analysis. Of the 146 enrolled, 139 were retained in the final analytic sample after excluding seven observations with biologically implausible BMI-for-age z-scores (see Section 2.2.1). The original study was approved on 2 February 2022 by the University of Cincinnati Institutional Review Board (#2021-0894). For reporting this study, STROBE guidelines were used [20].

### 2.2. Measures

#### 2.2.1. Caregiver and Child Characteristics

All demographic variables were included as covariates based on the prior literature and the SCT-guided conceptual framework. Caregivers self-reported age, sex, race, ethnicity, marital status, education, and perceived income adequacy. Race was dichotomized (White/non-White), ethnicity as Hispanic/non-Hispanic, and marital status as married/not married (due to small cell sizes, n ≤ 9, in remaining categories). Perceived income adequacy used three response options: comfortable, just enough, or not enough. Caregiver BMI was calculated from self-reported height and weight.

Child age, sex, height, and weight were caregiver-reported. Child BMI was converted to age- and sex-specific BMI-for-age z-scores using CDC 2000 growth chart LMS parameters, with each child’s age mapped to the nearest available LMS reference age in months. Z-scores were screened for biologically implausible values (<−4 SD). Seven observations fell below this threshold and were excluded (final analytic n = 139). These likely reflected caregiver data entry errors. Weight status was classified using standard CDC BMI-for-age percentile cut-points as the following: underweight (<5th percentile), healthy weight (≥5th to <85th percentile), overweight (≥85th to <95th percentile), and obesity (≥95th percentile) [21,22]. Continuous BMI-for-age z-score was used as a covariate in multivariable models.

#### 2.2.2. Caregiver Perception of Child Weight

In this study, perception was defined as a cognitive appraisal of how caregivers categorize their child’s current weight status and may be accurate or misaligned with BMI-based classifications. Perception was assessed with a single item (“How do you consider the weight of your child?”) with options: underweight, normal weight, overweight, and obese, consistent with prior parental perception research [23]. “Normal weight” was treated as equivalent to the CDC “healthy weight” category. Accuracy was classified by comparing perceived to measured BMI-for-age category: accurate (match), underestimation (perceived lower), or overestimation (perceived higher). Where cell frequencies were sparse (n < 10), overweight and obesity categories were combined prior to classification, consistent with prior practice [23,24]. All classification rules were established a priori and applied consistently across the sample.

#### 2.2.3. Dependent Variable: Caregiver Feeding Practices

Feeding practices were assessed using four subscales from the Comprehensive Feeding Practices Questionnaire (CFPQ) [25]. The CFPQ assesses multiple dimensions of feeding behavior for children aged approximately 2–8 years. The four subscales were (1) pressure to eat, (2) restriction for health, (3) restriction for weight control, and (4) monitoring. Items were scored on a 5-point Likert scale and averaged within each subscale, yielding scores ranging from 1 to 5. Internal consistency in the present sample (n = 139) was calculated, showing acceptable to good across all four subscales as follows: pressure to eat (α = 0.78), restriction for health (α = 0.86), restriction for weight control (α = 0.88), and monitoring (α = 0.88). These subscales represent related but conceptually distinct dimensions of caregiver feeding behavior.

#### 2.2.4. Statistical Analysis

All analyses were conducted using Stata/SE 18 (StataCorp LLC., College Station, TX, USA) [26]. Descriptive analyses characterized the sample overall and by child weight-status category and caregiver weight-perception group (accurate perception, underestimation, overestimation). Continuous variables were summarized as means and standard deviations, and categorical variables as frequencies and percentages. Cronbach’s alpha was calculated to evaluate the internal consistency of each feeding practice subscale within the present sample (see Section 2.2.3).

Separate multivariable linear regression models were fit for each feeding practice subscale (pressure to eat, restriction for health, restriction for weight control, and monitoring). Each subscale was modeled both unadjusted (Model 1) and adjusted (Model 2). The models estimated the independent association between weight-perception group (reference: accurate perception) and the subscale. This approach was guided by the study’s SCT-guided conceptual framework (Figure 1). Each adjusted model included child BMI-for-age z-score (continuous), child age, child sex, caregiver BMI, caregiver age, race, perceived income adequacy, education, and marital status as covariates, identified a priori from the prior literature. Child BMI-for-age z-score was retained as a covariate because it functions simultaneously as a determinant of misperception group membership and an independent predictor of feeding practices, satisfying the criteria for confounding. Including BMI z-score allows estimation of the association between weight misperception and feeding practices independent of the child’s actual weight. This independent association is the primary inferential target of this study. To assess sensitivity to this analytical choice, we refit all adjusted models excluding BMI z-score while retaining all other covariates (Supplementary Table S1). Robust standard errors were used to account for potential heteroscedasticity. Regression assumptions, including linearity, homoscedasticity, normality of residuals, and absence of multicollinearity, were assessed using residual plots and variance inflation factors (VIF). Analyses were based on complete cases, as missingness on key variables was minimal; no multiple imputation was performed. Because four CFPQ subscales were examined as separate outcomes against the same exposure, we did not apply a formal correction for multiple comparisons. The four outcomes were pre-specified based on the SCT-guided conceptual framework. Although they are correlated subscales from the same instrument, they represent conceptually distinct feeding behaviors pre-specified as independent outcomes. The study is explicitly exploratory and hypothesis-generating; applying conservative corrections in this context would substantially increase Type II error risk. All *p*-values, 95% confidence intervals, and effect sizes are reported to support magnitude-based interpretation. Statistical significance was set at *p* < 0.05 (two-sided), and regression coefficients with 95% confidence intervals were reported to convey the direction and magnitude of associations.

## 3. Results

### 3.1. Descriptive Statistics

Table 1 presents the characteristics of the analytic sample (N = 139). Caregivers had a mean age of 35.47 years (SD = 6.33), mean BMI of 32.46 kg/m^2^ (SD = 9.54), and mean education of 16.37 years (SD = 4.13); most identified as White (77.0%), were married (76.3%), and perceived their income as comfortable or just sufficient (92.8%).

Children had a mean age of 3.88 years (SD = 0.80), were approximately equally distributed by sex (51.1% female), and had a mean BMI-for-age z-score of 0.54 (SD = 1.57). Based on CDC classifications, 41.0% had overweight or obesity. Caregivers reported moderate use of pressure to eat (M = 2.46, SD = 0.97) and restriction for weight control (M = 1.54, SD = 0.71), and higher use of restriction for health (M = 3.33, SD = 1.22) and monitoring (M = 3.63, SD = 1.05), all on a 1–5 scale.

Table 2 presents the distribution of caregiver weight-perception group by child weight status. Overall, 45.3% of caregivers accurately perceived their child’s weight, 44.6% underestimated it, and 10.1% overestimated it. Misperception was strongly patterned by child weight status. All caregivers of children with overweight (100%) and most of those with obesity (90.6%) underestimated their child’s weight status. In contrast, all caregivers of underweight children (100%) overestimated it. Accurate perception was most common among caregivers of healthy-weight children (88.2%). Among caregivers in the underestimation group (n = 62), 82.3% (n = 51) classified their child as ‘normal weight’ and 17.7% (n = 11) classified their child as ‘underweight’ (χ^2^ = 14.84, *p* = 0.001). All caregivers in the overestimation group (n = 14) classified their child as ‘normal weight.’ Thus, overestimation in this sample exclusively reflected upward misclassification of normal-weight children.

### 3.2. Multivariable Associations Between Caregiver Weight-Perception Group and Feeding Practices

Table 3 presents the unadjusted and adjusted associations between caregiver weight-perception group and each of the four feeding practice subscales. In unadjusted models, weight-perception group was not significantly associated with any feeding practice subscale (all *p* > 0.05). In adjusted models, underestimation of child weight was independently associated with lower restrictive feeding, relative to caregivers with accurate perception. This applied to both restriction for health (B = −0.62, 95% CI: −1.10, −0.13, *p* = 0.013) and restriction for weight control (B = −0.30, 95% CI: −0.58, −0.02, *p* = 0.033). The observed standard deviations were 1.22 for restriction for health and 0.71 for restriction for weight control. Relative to these, the coefficients correspond to roughly one-half and two-fifths of a standard deviation, respectively. These represent moderate differences in restrictive feeding practices associated with underestimation. Overestimation was associated with higher restriction for weight control at the margin of statistical significance (B = 0.53, 95% CI: 0.00, 1.07, *p* = 0.050). No significant associations were observed between weight-perception group and pressure to eat or monitoring in either model. Any observed coefficient differences for these subscales were small (<0.20 points, <0.3 SD). They are therefore best interpreted as modest variations in feeding behavior.

Among covariates, child BMI-for-age z-score was positively associated with restriction for health (B = 0.19, 95% CI: 0.01, 0.37, *p* = 0.037) and restriction for weight control (B = 0.20, 95% CI: 0.08, 0.33, *p* = 0.001), indicating that caregivers of children with higher BMI reported greater use of both restrictive practices. Child age was positively associated with restriction for health (B = 0.24, 95% CI: 0.01, 0.47, *p* = 0.043). Caregiver education was inversely associated with three subscales: restriction for health (B = −0.07, 95% CI: −0.12, −0.02, *p* = 0.004), restriction for weight control (B = −0.02, 95% CI: −0.04, −0.002, *p* = 0.031), and monitoring (B = −0.07, 95% CI: −0.12, −0.03, *p* = 0.001). In other words, caregivers with more years of education reported lower use of these feeding practices. The adjusted model explained the greatest variance in restriction for health (R^2^ = 0.187, Adjusted R^2^ = 0.109), followed by restriction for weight control (R^2^ = 0.150, Adjusted R^2^ = 0.069) and monitoring (R^2^ = 0.128, Adjusted R^2^ = 0.045), while explaining minimal variance in pressure to eat (R^2^ = 0.054, Adjusted R^2^ = −0.036). The addition of covariates in Model 2 produced a statistically significant improvement in model fit for restriction for health (R^2^ change = 0.179, *p* = 0.004) and restriction for weight control (R^2^ change = 0.146, *p* = 0.023), but not for pressure to eat (*p* = 0.880) or monitoring (*p* = 0.089). In sensitivity analyses excluding child BMI-for-age z-score, the associations between weight-perception group and restrictive feeding practices were attenuated and no longer statistically significant, consistent with positive confounding by actual child weight being reintroduced when BMI z-score is omitted (Supplementary Table S1).

## 4. Discussion

This study examined whether caregiver weight perception was independently associated with four feeding practice subscales among primary caregivers of preschoolers in the Midwestern U.S. Results showed that caregiver weight misperception was prevalent and independently associated with restrictive feeding practices, but not with pressure to eat or monitoring. After full adjustment for child and caregiver characteristics, underestimation was independently associated with lower use of restriction for health and restriction for weight control. Overestimation, by contrast, was marginally associated with higher restriction for weight control. The size of these differences was consistent with small-to-moderate shifts on the 1–5 CFPQ scale, suggesting that misperception is associated with meaningful but not extreme variation in restrictive feeding. These findings advance understanding of how perception-driven cognitions may selectively associate with specific feeding behaviors in early childhood.

### 4.1. Prevalence of Weight Misperception

The prevalence of weight underestimation observed in this study (44.6%) is consistent with the prior literature, particularly if interpreted in relation to children with overweight or obesity. A meta-analysis of 69 studies including 15,791 children with overweight or obesity found that 50.7% of parents underestimated their child’s weight status [14]. Similar patterns have been documented across countries. In China, more than one-third of primary caregivers underestimated their child’s weight status overall, and more than half of caregivers of children with overweight or obesity underestimated their child’s weight [27]. In a high-deprivation community in New Zealand, only 16% of children with overweight or obesity were correctly perceived by caregivers as having overweight [28]. Similarly, a Dutch population-based study found that approximately 61–65% of parents underestimated the weight status of children with overweight, with even higher underestimation among children with obesity [29]. These cross-national findings highlight the widespread nature of caregiver misperception.

One likely explanation is the normalization of higher body weight within social reference groups, particularly in communities where childhood overweight and obesity are common [28]. Limited alignment between caregiver perceptions and clinical BMI classifications may also contribute to misperception [27,29]. Importantly, these misperceptions are not benign. Maternal perception, concern, and dissatisfaction with child weight have been associated with feeding practices such as restriction, covert control, and pressure to eat, even after accounting for actual child weight status [6]. Similarly, a systematic review and meta-analysis found that caregiver concern about child overweight was positively associated with restrictive feeding, while concern about child underweight was associated with greater pressure to eat [10]. Overall, these findings suggest that how caregivers interpret and emotionally respond to their child’s weight, not only the child’s measured weight status, may shape feeding behaviors. This underscores the importance of addressing perceptual and cognitive factors in family-based obesity prevention and treatment interventions.

### 4.2. Associations Between Weight Misperception and Feeding Practices

The absence of significant associations in unadjusted models and their emergence after covariate adjustment warrants explanation. This pattern reflects positive confounding, primarily by child BMI-for-age z-score. Children with higher BMI z-scores are disproportionately represented in the underestimation group, and they independently receive more restrictive feeding. As a result, unadjusted estimates are biased toward the null. The true negative misperception–restriction association is masked by the positive effect of actual child weight. After adjusting for BMI z-score, this confounding is removed and the independent association between misperception and restrictive feeding becomes apparent. Because misperception is operationally defined by the discordance between caregiver-perceived and BMI-based weight category, child BMI-for-age z-score is conceptually linked to the exposure. We treated it as a confounder rather than a mediator, because actual child weight plausibly precedes and shapes both caregiver perception and feeding behavior, rather than lying on a causal pathway from misperception to feeding practices; conditioning on it therefore isolates the association attributable to perception itself rather than to the child’s measured weight. This interpretation is corroborated by sensitivity analyses excluding BMI z-score (Supplementary Table S1), in which the adjusted associations attenuated to non-significance, mirroring the unadjusted Model 1 results. Caregiver education, which was inversely associated with restrictive feeding practices across multiple subscales, contributed secondarily to the change in estimates between models.

In the present study, parental weight underestimation was associated with lower restrictive feeding. This suggests that caregivers who perceive their child as being in a lower weight category than their measured status may be less likely to limit the child’s food intake. This finding is consistent with prior evidence showing that parents’ interpretations of child weight are linked to feeding practices. Although relatively few studies have examined misperception using the same underestimation and overestimation categories as the present study, studies of related constructs provide useful context. Chen et al. (2025), in a prospective longitudinal study of Chinese preschoolers, found that parental perception of the child as underweight was associated with lower restrictive feeding in generalized estimating equation models (B = −0.260, *p* = 0.004) [5]. Similarly, Costa et al. (2021) reported that perceived underweight was associated with lower restriction at age 4 years in the Generation XXI birth cohort [6]. These findings support the interpretation that caregivers who view a child as smaller or lighter are less likely to use intake-limiting feeding practices. The present study extends this literature by focusing on weight-status misperception rather than perceived weight category alone. Because underestimation was associated with lower restriction whether framed around health or weight control, it may reflect a broad reduction in intake-limiting behavior rather than a narrower tendency to restrict food specifically for weight control.

The marginal association between parental overestimation of child weight status and greater restriction for weight control, but not restriction for health, suggests that overestimation may be linked specifically to weight-focused feeding responses. Parents who perceive their child as heavier than their measured weight status may be more likely to limit foods with the explicit goal of controlling weight, rather than for broader health-promotion reasons. This distinction is important because restriction motivated by weight control may reflect parental concern about excess weight, body size, or future weight gain, whereas health-oriented restriction may reflect more general beliefs about healthy eating.

Although few studies have examined overestimation using the same misperception categories as the present study, related evidence supports the plausibility of this pattern. Xiang et al. (2021) reported that parental concern about overweight was associated with higher restriction among normal-weight children [30], a subgroup in which overestimation is especially likely to occur. Similarly, Wang et al. (2022) found that caregiver concern about child overweight was positively associated with restrictive feeding across studies [10]. These studies do not provide direct evidence on overestimation, because concern about overweight is distinct from misperception. However, they support the broader interpretation that caregiver cognitive and emotional appraisal of excess weight may contribute to restrictive feeding.

The marginal and potentially unstable nature of the overestimation finding warrants considerable caution. With only 14 caregivers in the overestimation group, the study was underpowered to detect associations reliably for this subgroup, and the wide confidence interval (0.00, 1.07) indicates high uncertainty around the point estimate. Overestimation is also less common than underestimation in populations with higher overweight prevalence, further constraining power. This finding should be considered preliminary and hypothesis-generating, pending replication in larger samples. In addition, the behavioral effects of overestimation may be concentrated among children with normal weight, because these children are most likely to be incorrectly perceived as having overweight. Hu et al. (2022) found that associations between parental weight perceptions and feeding practices were significant primarily among normal-weight children, supporting the possibility that overestimation-related feeding responses may be most visible in this subgroup [31].

In contrast, neither underestimation nor overestimation was significantly associated with pressure to eat or monitoring in adjusted models. Although previous studies have found that caregivers who perceive their child as underweight are more likely to pressure the child to eat [5,6], this pattern did not emerge in the present study. A plausible explanation is that many caregivers who underestimated their child’s weight may have classified the child as normal weight rather than underweight. Such misperception may be associated with lower motivation to restrict intake but may not generate concern about inadequate intake, which appears to be a more proximal driver of pressure-to-eat behaviors. Brown et al. (2016) found that maternal concern for child undereating was associated with greater reported pressure to eat and with observed pressuring and bribery during meals, and that this concern was more strongly related to picky eating and lower child BMI-z than to weight perception alone [32]. Thus, pressure to eat may be driven less by weight-status misclassification itself and more by specific concerns about inadequate intake or child eating behavior. Similarly, monitoring was not significantly associated with either underestimation or overestimation. This suggests that monitoring may be less responsive to weight misperception than restriction. Prior evidence indicates that monitoring may reflect broader parenting orientation or general concern rather than misclassification of child weight status alone. Loth et al. (2021) found that weight-related concern was associated with monitoring, while van der Horst and Sleddens (2017) linked monitoring to broader parenting and feeding-style patterns [33,34]. Therefore, the absence of an association between misperception and monitoring in the present study may indicate that monitoring is influenced more by general parenting tendencies and explicit concern than by whether the child’s weight status is accurately classified.

Overall, the findings suggest a domain-specific association between weight misperception and feeding practices. Misperception was most clearly associated with restrictive feeding, particularly lower restriction among caregivers who underestimated child weight, whereas pressure to eat and monitoring appeared less closely tied to weight misclassification. This specificity is important for intervention design. Correcting caregiver misperception may be relevant for addressing misperception-driven changes in restrictive feeding, but it may be insufficient to change other feeding practices unless interventions also address the distinct concerns, beliefs, and parenting patterns that drive pressure to eat and monitoring. Whether correcting misperception would actually change feeding behavior cannot be confirmed from cross-sectional data and remains to be tested.

### 4.3. Strengths, Limitations, and Generalizability

This study is among the few U.S.-based studies to examine associations between parental weight misperception and feeding practices in preschool-aged children. The study was guided by Social Cognitive Theory, which provides a framework for understanding how parental perceptions may influence feeding practices. Additionally, caregiver weight perception was classified relative to CDC categories, and feeding practices were assessed using four validated CFPQ subscales with good internal consistency (α = 0.78–0.88). Importantly, social media platforms such as Facebook were used as a primary recruitment method in this study, which may have facilitated access to a broader and more geographically diverse sample of caregivers.

However, the results of this study should be interpreted with caution due to several limitations. First, the cross-sectional nature of the study limits the ability to determine the directionality or causality of the observed associations. Second, data were collected via self-reported questionnaires, which may introduce reporting bias and social desirability bias. Specifically, this may affect BMI-for-age classification accuracy and directly impacts the definition of weight misperception. More specifically, because the misperception classification is derived by comparing caregiver-perceived weight with BMI-based category, over- or under-reporting of child height or weight could misclassify a child’s BMI category and, in turn, the perception group to which the caregiver is assigned; this represents potential misclassification of the core exposure itself, not only attenuation of the observed associations. Parental underreporting of child weight is well-documented and would tend to attenuate associations toward the null, suggesting that our significant findings are conservative estimates. Third, caregiver weight perception was assessed using a single-item measure, which may not fully capture the complexity of perceptual processes. Additionally, the study has a modest sample size (n = 139), particularly the small number of caregivers in the overestimation group (n = 14), which severely limited statistical power for this subgroup. The borderline significance (*p* = 0.050) and wide confidence interval (0.00, 1.07) for the overestimation–restriction for weight control association suggest the estimate is unstable and should not be interpreted as reliable evidence of an effect without replication in a larger, more balanced sample. Finally, the use of Facebook advertising and community-based convenience sampling may have introduced self-selection bias. Caregivers who chose to participate were likely more digitally connected, health-engaged, and socioeconomically advantaged than the broader population of U.S. caregivers of preschool-aged children. This may have biased upward the prevalence of accurate perception and certain feeding practices, while underrepresenting caregivers from racially, ethnically, and socioeconomically diverse backgrounds. Consistent with this, the sample was predominantly White (77.0%), married (76.3%), and college-educated (mean = 16.4 years), which may limit generalizability to more diverse populations where weight misperception and its behavioral consequences may differ. In addition, residual confounding is likely. We did not measure several factors that may influence both caregiver weight perceptions and feeding practices, including caregiver concern about child weight, child eating behavior (e.g., picky eating and appetite), food insecurity, and aspects of the home dietary environment. These unmeasured characteristics could partly account for the observed associations. More broadly, the significant associations were modest in magnitude and some approached the conventional significance threshold, so the overall pattern of findings should be regarded as preliminary. Our findings should therefore be interpreted as hypothesis-generating rather than definitive.

## 5. Conclusions

In this theory-driven cross-sectional study of U.S. caregivers of preschoolers, weight underestimation was independently linked to lower restriction for health and weight control, while overestimation was marginally associated only with higher restriction for weight control. No associations were observed with pressure to eat or monitoring, indicating that weight misperception is selectively associated with restrictive feeding rather than feeding practices broadly. Given that underestimation was most common, it was the misperception most often associated with lower use of restrictive feeding practices; however, given the mixed evidence on parental restriction and child dietary outcomes, the clinical implications of this pattern remain uncertain. These findings suggest that weight misperception may be a candidate cognitive target in obesity prevention research. However, cross-sectional data cannot establish whether correcting misperceptions would lead to changes in feeding behavior. Longitudinal or experimental studies are needed to test this possibility. Future longitudinal research with larger, more diverse samples and measured anthropometrics is needed to confirm causality and clarify subgroup-specific effects.

## Figures and Tables

**Table 1:** Sample characteristics of caregivers and children (N = 139).

Characteristic	n (%) or Mean ± SD
Caregivers	
Age (years)	35.47 ± 6.33
BMI (kg/m^2^)	32.46 ± 9.54
Education (years)	16.37 ± 4.13
Race	
White	107 (77.0%)
Non-White	32 (23.0%)
Marital status	
Married	106 (76.3%)
Member of unmarried couple	9 (6.5%)
Never married	16 (11.5%)
Divorced	8 (5.8%)
Perceived income adequacy	
Comfortable	63 (45.3%)
Just enough	66 (47.5%)
Not enough	10 (7.2%)
Children	
Age (years)	3.88 ± 0.80
Sex	
Male	68 (48.9%)
Female	71 (51.1%)
BMI (kg/m^2^)	17.83 ± 6.67
BMI-for-age z-score	0.54 ± 1.57
BMI category (CDC cut-points)	
Underweight	14 (10.1%)
Normal weight	68 (48.9%)
Overweight	25 (18.0%)
Obesity	32 (23.0%)
Perceived child weight status by caregiver	
Underweight	11 (7.9%)
Normal weight	125 (89.9%)
Overweight	2 (1.4%)
Obesity	1 (0.7%)
Weight Perception (vs. CDC BMI classification)	
Accurate perception	63 (45.3%)
Underestimation	62 (44.6%)
Overestimation	14 (10.1%)
Feeding Practice Subscales (range 1–5)	
Pressure to eat	2.46 ± 0.97
Restriction for health	3.33 ± 1.22
Restriction for weight control	1.54 ± 0.71
Monitoring	3.63 ± 1.05

Note: Percentages for categorical variables are based on the analytic sample (n = 139) and may not sum to exactly 100.0% because of rounding.

**Table 2:** Caregiver weight-perception group by child BMI classification and caregiver-perceived weight label (N = 139).

	Accurate Perception n (%)	Underestimation n (%)	Overestimation n (%)	Total n (%)
**Actual Child Weight Status (CDC BMI) ^a^**				
Underweight	0 (0.0%)	0 (0.0%)	14 (100.0%)	14 (100.0%)
Normal weight	60 (88.2%)	8 (11.8%)	0 (0.0%)	68 (100%)
Overweight	0 (0.0%)	25 (100.0%)	0 (0.0%)	25 (100%)
Obesity	3 (9.4%)	29 (90.6%)	0 (0.0%)	32 (100%)
Total	63 (45.3%)	62 (44.6%)	14 (10.1%)	139 (100%)
**Caregiver-Perceived Weight Label ^b^**				
Underweight	0 (0.0%)	11 (17.7%)	0 (0.0%)	11 (7.9%)
Normal weight	60 (95.2%)	51 (82.3%)	14 (100.0%)	125 (89.9%)
Overweight	2 (3.2%)	0 (0.0%)	0 (0.0%)	2 (1.4%)
Obesity	1 (1.6%)	0 (0.0%)	0 (0.0%)	1 (0.7%)
Total	63 (100.0%)	62 (100.0%)	14 (100.0%)	139 (100.0%)

Note: Weight-perception group was defined by comparing caregiver-perceived weight label to CDC BMI-for-age classification. Percentages are row percentages in Panel A and column percentages in Panel B. ^a^ Pearson χ^2^(6) = 18.04, *p* = 0.006. ^b^ Pearson χ^2^(6) = 18.04, *p* = 0.006. Percentages for categorical variables are based on the analytic sample (n = 139) and may not sum to exactly 100.0% because of rounding.

**Table 3:** Unadjusted and adjusted associations between caregiver weight-perception group and feeding practices (N = 139).

Predictor	Pressure to Eat	Restriction for Health	Restriction for Weight Control	Monitoring
	β (95% CI)	β (95% CI)	β (95% CI)	β (95% CI)
Model 1—Unadjusted				
Weight-Perception Group (ref: Accurate perception)				
Underestimation	0.24 (−0.11, 0.59)	−0.06 (−0.50, 0.37)	0.04 (−0.21, 0.29)	0.18 (−0.19, 0.54)
Overestimation	0.26 (−0.22, 0.75)	0.30 (−0.41, 1.00)	0.15 (−0.30, 0.60)	0.30 (−0.39, 0.99)
Model Fit				
F (2, 136)	1.12	0.49	0.23	0.66
*p*-value	0.330	0.611	0.797	0.516
R^2^	0.016	0.007	0.004	0.010
Model 2—Adjusted				
Weight-Perception Group (ref: Accurate perception)				
Underestimation	0.28 (−0.14, 0.70)	−0.62 (−1.10, −0.13) **	−0.30 (−0.58, −0.02) **	−0.02 (−0.52, 0.49)
Overestimation	−0.10 (−0.76, 0.56)	0.62 (−0.22, 1.47)	0.53 (0.00, 1.07) *	0.17 (−0.67, 1.00)
Child Characteristics				
BMI-for-age z-score	−0.09 (−0.26, 0.07)	0.19 (0.01, 0.37) **	0.20 (0.08, 0.33) ***	0.02 (−0.16, 0.19)
Age (years)	0.02 (−0.20, 0.24)	0.24 (0.01, 0.47) **	0.05 (−0.12, 0.22)	0.11 (−0.12, 0.34)
Sex (ref: Female)	−0.09 (−0.44, 0.25)	0.12 (−0.29, 0.53)	0.11 (−0.14, 0.37)	−0.02 (−0.38, 0.34)
Caregiver Characteristics				
BMI (kg/m^2^)	0.01 (−0.01, 0.02)	0.00 (−0.02, 0.02)	0.004 (−0.01, 0.02)	−0.002 (−0.02, 0.02)
Age (years)	−0.004 (−0.03, 0.02)	−0.005 (−0.03, 0.02)	0.009 (−0.01, 0.03)	0.009 (−0.02, 0.04)
Education (years)	0.01 (−0.03, 0.05)	−0.07 (−0.12, −0.02) ***	−0.02 (−0.04, −0.002) **	−0.07 (−0.12, −0.03) ***
White (ref: Non-White)	−0.18 (−0.62, 0.26)	−0.38 (−0.90, 0.14)	−0.27 (−0.62, 0.09)	−0.31 (−0.76, 0.13)
Marital status (married)	−0.09 (−0.54, 0.36)	−0.42 (−0.92, 0.09)	0.12 (−0.21, 0.45)	−0.25 (−0.70, 0.20)
Perceived Income Adequacy (ref: Comfortable)				
Just have enough to make ends meet	−0.28 (−0.66, 0.10)	−0.06 (−0.54, 0.43)	0.03 (−0.25, 0.30)	−0.37 (−0.80, 0.05)
Do NOT have enough to make ends meet	−0.36 (−1.07, 0.34)	0.15 (−0.50, 0.79)	−0.25 (−0.63, 0.14)	−0.19 (−0.79, 0.40)
Model Fit				
F (12, 126)	0.72	3.24	1.80	1.80
*p*-value	0.732	<0.001	0.054	0.055
R^2^	0.054	0.187	0.150	0.128
Adjusted R^2^	−0.036	0.109	0.069	0.045
R^2^ change	0.038	0.179	0.146	0.118

Note: B = unstandardized regression coefficient; 95% CI = 95% confidence interval. Reference category for weight-perception group = accurate perception. Reference category for income adequacy = comfortable. Feeding practice subscales scored 1–5 (mean item scores). Robust standard errors applied to all models. Marital status was dichotomized as married vs. not married in multivariable models due to sparse cell sizes in remaining categories (n ≤ 9 per group). * *p* = 0.050; ** *p* < 0.05; *** *p* < 0.01.

## Data Availability

The data supporting the reported results of this study are not publicly available due to ethical restrictions and the protection of participant confidentiality. Access to the data may be considered on a case-by-case basis and can be obtained by contacting the corresponding author at qoudat@mailbox.sc.edu or oudatqh@mail.uc.edu.

## Supplementary Materials

The following supporting information can be downloaded at: https://www.mdpi.com/article/10.3390/nu18121880/s1, Table S1. Sensitivity analyses of associations between caregiver weight-perception group and feeding practices without adjustment for child BMI-for-age z-score (N = 139).

## Abbreviations

The following abbreviations are used in this manuscript:

CDC	Centers for Disease Control and Prevention
BMI	Body Mass Index
SCT	Social Cognitive Theory
CFPQ	Comprehensive Feeding Practices Questionnaire
REDCap	Research Electronic Data Capture
