# Supplementary material for: Caregiver Weight Misperception and Feeding Practices in U.S. Preschool-Aged Children: A Theory-Based Cross-Sectional Study
Source: Nutrients. 2026 Jun 11;18(12):1880. doi: 10.3390/nu18121880 (PMC13305503; doi:10.3390/nu18121880)
Supplement: Supplementary file 1 [file nutrients-18-01880-s001.zip › nutrients-4355774-supplementary.pdf]

Supplementary Table S1. Sensitivity analyses of associations between caregiver weight-perception group and feeding practices without adjustment for child BMI-for-age z-score (N = 139).

Predictor	Pressure to Eat β (95% CI)	Restriction for Health β (95% CI)	Restriction for Weight Control β (95% CI)	Monitoring β (95% CI)
**Weight-Perception Group (ref: Accurate perception)**				
Underestimation	0.16 (−0.22, 0.55)	−0.38 (−0.82, 0.07)	−0.05 (−0.27, 0.18)	0.01 (−0.44, 0.45)
Overestimation	0.14 (−0.37, 0.66)	0.11 (−0.63, 0.85)	−0.02 (−0.53, 0.50)	0.12 (−0.56, 0.80)
**Child Characteristics**				
Age (years)	0.03 (−0.19, 0.25)	0.22 (−0.02, 0.46)	0.03 (−0.15, 0.21)	0.11 (−0.12, 0.35)
Sex: Male (ref: Female)	−0.12 (−0.46, 0.22)	0.18 (−0.23, 0.58)	0.18 (−0.10, 0.45)	−0.02 (−0.37, 0.33)
**Caregiver Characteristics**				
BMI (kg/m^2)	0.009 (−0.008, 0.025)	0.000 (−0.020, 0.020)	0.004 (−0.010, 0.018)	−0.002 (−0.021, 0.017)
Age (years)	−0.003 (−0.030, 0.024)	−0.008 (−0.035, 0.019)	0.006 (−0.013, 0.025)	0.008 (−0.025, 0.041)
Education (years)	0.009 (−0.032, 0.050)	−0.069 (−0.118, −0.021) **	−0.018 (−0.042, 0.005)	−0.073 (−0.117, −0.030) ***
White (ref: Non-White)	−0.16 (−0.59, 0.28)	−0.43 (−0.94, 0.08)	−0.32 (−0.70, 0.06)	−0.32 (−0.75, 0.12)
Marital status (married)	−0.05 (−0.50, 0.40)	−0.50 (−1.01, 0.02)	0.03 (−0.28, 0.35)	−0.26 (−0.70, 0.19)
**Perceived Income Adequacy (ref: Comfortable)**				
Just have enough to make ends meet	−0.26 (−0.64, 0.12)	−0.10 (−0.59, 0.40)	−0.02 (−0.31, 0.28)	−0.38 (−0.80, 0.05)
Do NOT have enough to make ends meet	−0.35 (−1.08, 0.38)	0.12 (−0.50, 0.74)	−0.28 (−0.68, 0.12)	−0.20 (−0.80, 0.40)
**Model Fit**				
F (11, 127)	0.59	3.13	1.09	1.97
p-value	0.836	<.001	0.377	0.036
R^2	0.046	0.164	0.073	0.128

Note. β = unstandardized regression coefficient; 95% CI = 95% confidence interval. Reference category for weight-perception group = accurate perception. Reference category for income adequacy = comfortable. Child BMI-for-age z-score was excluded from all models; all other covariates from the primary adjusted models (Table 3) were retained. Feeding practice subscales scored 1–5 (mean item scores). Robust standard errors applied. Marital status dichotomized as married vs. not married due to sparse cell sizes in remaining categories ($n \leq 9$ per group). ** $p < .01$; *** $p < .001$.
